# In Situ, High-Resolution Profiles of Labile Metals in Sediments of Lake Taihu

**DOI:** 10.3390/ijerph13090884

**Published:** 2016-09-06

**Authors:** Dan Wang, Mengdan Gong, Yangyang Li, Lv Xu, Yan Wang, Rui Jing, Shiming Ding, Chaosheng Zhang

**Affiliations:** 1 State Key Laboratory of Lake Science and Environment, Nanjing Institute of Geography and Limnology, Chinese Academy of Sciences, 73 East Beijing Road, Nanjing 210008, China; wangdan213@mails.ucas.ac.cn (D.W.); gongmengdan14@mails.ucas.ac.cn (M.G.); 15051895212@163.com (Y.L.); yuyan19881116@163.com (Y.W.); rjing@niglas.ac.cn (R.J.); 2 University of Chinese Academy of Sciences, 19 A Yuquan Road, Beijing 100039, China; 3 School of Chemical Engineering, Nanjing University of Science and Technology, 200 Xiaolingwei Road, Nanjing 210094, China; 4 Key Laboratory of Integrated Regulation and Resource Development on Shallow Lakes, Ministry of Education, College of Environment, Hohai University, 1 Xikang Road, Nanjing 210098, China; hhuxulv@126.com; 5 International Network for Environment and Health, School of Geography and Archaeology, National University of Ireland, Galway, University Road, Galway H91 CF50, Ireland; Zhang@nuigalway.ie

**Keywords:** metals, high resolution, diffusive gradient in thin films, relative mobility, sediment, in-situ

## Abstract

Characterizing labile metal distribution and biogeochemical behavior in sediments is crucial for understanding their contamination characteristics in lakes, for which in situ, high-resolution data is scare. The diffusive gradient in thin films (DGT) technique was used in-situ at five sites across Lake Taihu in the Yangtze River delta in China to characterize the distribution and mobility of eight labile metals (Fe, Mn, Zn, Ni, Cu, Pb, Co and Cd) in sediments at a 3 mm spatial resolution. The results showed a great spatial heterogeneity in the distributions of redox-sensitive labile Fe, Mn and Co in sediments, while other metals had much less marked structure, except for downward decreases of labile Pb, Ni, Zn and Cu in the surface sediment layers. Similar distributions were found between labile Mn and Co and among labile Ni, Cu and Zn, reflecting a close link between their geochemical behaviors. The relative mobility, defined as the ratio of metals accumulated by DGT to the total contents in a volume of sediments with a thickness of 10 mm close to the surface of DGT probe, was the greatest for Mn and Cd, followed by Zn, Ni, Cu and Co, while Pb and Fe had the lowest mobility; this order generally agreed with that defined by the modified BCR approach. Further analyses showed that the downward increases of pH values in surface sediment layer may decrease the lability of Pb, Ni, Zn and Cu as detected by DGT, while the remobilization of redox-insensitive metals in deep sediment layer may relate to Mn cycling through sulphide coprecipitation, reflected by several corresponding minima between these metals and Mn. These in situ data provided the possibility for a deep insight into the mechanisms involved in the remobilization of metals in freshwater sediments.

## 1. Introduction

Heavy metals in aquatic ecosystems come mainly from Nature (background levels) and anthropogenic activities [1]. Over the past few decades, there has been an increase in the discharge of untreated or partially treated sewage from industry, agriculture, and urban life. The resulting deposition of specific heavy metals has contributed to significant heavy metals pollution [2,3,4]. Heavy metals pose risks to aquatic ecosystems, because of their toxicity and non-degradability [5]. Furthermore, the biological accumulation of heavy metals by aquatic fauna and flora increases health risks for human beings, especially for the kidney, liver, circulatory systems, and nerve tissue [6,7].

Sediments are important components of aquatic ecosystems, acting as a both a sink and source of heavy metals and other pollutants. Even a small change in sediments, such as variations in pH, Eh, or dissolved oxygen (DO), can release heavy metals from the solid to the solution phase, or release metals from sediments into the overlying water [8,9]. Given this, it is important to research the biogeochemical behavior of heavy metals in sediments.

Lake Taihu, the third largest freshwater lake in China, located on the Yangtze River delta, suffers from eutrophication and heavy metals contamination as a result of population expansion and heavy industrial and agricultural expansion [10,11]. Lake Taihu has experienced differences in water quality and ecology [12,13]. For example, Cu, Ni, Zn contamination, resulting from human activities, was found in the north Meiliang and Zhushan Bays; Pb is higher in southwest and east lake areas [14]. Previous research has focused on the total metal content distribution in Lake Taihu sediments and ecological risk assessment based on those total concentrations [15,16,17,18]. However, high total metal concentrations are not necessarily significantly toxic for benthic organisms [19,20]. Heavy metals are mainly bound to sediments through adsorption, coordination, and precipitation, which reduces their bioavailability [21,22]. Ultimately, it is the labile fractions of heavy metals, rather than total concentrations, that most impact toxicity and bioavailability [23,24].

Previous studies on the lability of heavy metals in sediments have primarily applied two ex-situ techniques. The first technique involves slicing sediment cores, and then measuring dissolved metals in the pore water collected from the cores. Metal flux through the sediment-water interface (SWI) is calculated based on the dissolved metal distributions near the SWI. It is best to collect pore water samples in a high purity nitrogen gas environment; however, it is hard to avoid the disturbance of oxidation environments and changes in metal speciation. Further, the spatial resolution of slices (~1 cm) fail to reflect the heterogeneous distribution of metals in surface sediments, and pore water measurements do not reflect the kinetic exchange of metals between the solid phase and the pore water [1].

The second technique involves the extraction of labile metals using chemical fractionation techniques [25]. A number of single or sequential extraction methods have been built and introduced in related studies. Tessier’s procedure and European Communities Bureau of References (BCR) are two widely accepted procedures [26,27,28]. These are operationally defined methodologies, and there remains some debate as to whether the metals extracted in different leaching liquors accurately reflect the metal mobility and bioavailability [19,21,29].

Diffusive gradients in thin films (DGT), developed by Davison and Zhang, is an in-situ, dynamic, and high-resolution technique [30,31] that allows the study of the distribution and mobility of labile metals in sediments. The DGT method is grounded in Fick’s First Law of Diffusion. It measures the accumulation mass or mean concentration of metals during deployment in sediment, which comes from pore water and the further resupply of metals from solid phase [32,33]. Labile metals pass the diffusion layer and are then immobilized in the binding layer, which consists of free metal ions, metal ions present as simple inorganic complexes, and labile organic complexes [34].

DGT measurements simulate the kinetic exchange of labile metal species from the decrease in dissolved metals in pore water, because of biological uptake or similar abiotic perturbation [35]. Hence, DGT is a better tool for characterizing dynamical distribution and mobility of metals than traditional methods. Previous DGT studies on metals, such as Fe, Mn, Zn, Ni, Cu, Pb, Co and Cd, were mostly limited to laboratory tests [8,20,24,36]; in situ deployment was scarce.

In this study, the DGT probe was used in-situ to measure the labile fractions (reflected by DGT fluxes) of eight metals (Fe, Mn, Zn, Ni, Cu, Pb, Co and Cd) in sediments from five sites in Lake Taihu at a vertical resolution of 3 mm. The overall lability of these metals in sediments was assessed based on the DGT measurements. The apparent fluxes of these metals from sediments were calculated based on the DGT profiles. The mechanisms responsible for the remobilization of these metals in sediments were further revealed by relating the distributions of DGT-labile fractions to potentially governing factors.

## 2. Material and Methods

### 2.1. Description of Sampling Sites

**Figure 1:**
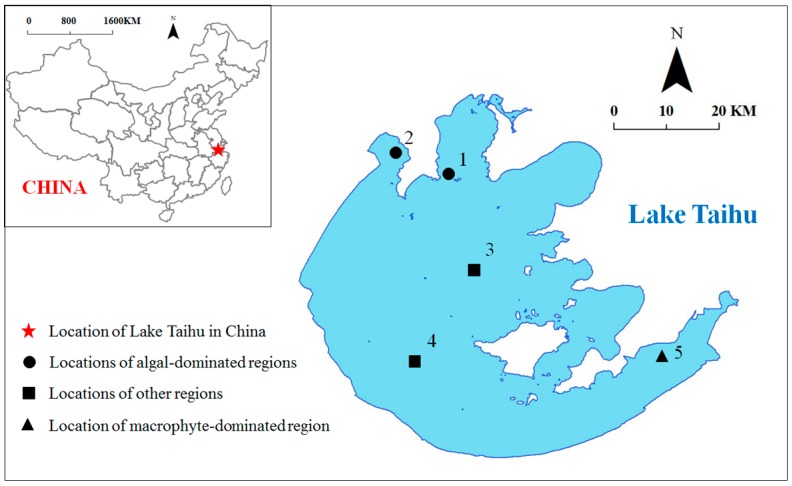
Locations of Lake Taihu in China and sampling sites in Lake Taihu.

Figure 1 shows the five Lake Taihu sites selected for this study; samples were collected in October 2014. These sites can be classified into three ecological types (Table 1). Sites 1 and 2 are located in algal-dominated regions (Meiliang and Zhushan Bays) with polluted characteristics. Site 5 is located in the macrophyte-dominated region (East Lake Taihu); this is an aquaculture region with fresh aquatic vegetation and small aquatic animals in the water column which feed crabs. Sites 3 and 4 are in the central and west part of the lake, respectively. There is no obvious macrophyte coverage in the two site regions, but they frequently suffer from algal blooms.

### 2.2. DGT Preparation and Field Deployment

In a preliminary experiment, we used a typical DGT probe with a 0.90 mm-thickness diffusion layer to measure metals in Lake Taihu at a vertically spatial resolution of 3 mm, with a typical deployment time of 24 h. However, several trace metals could not be detected due to low masses accumulated by DGT. Consequently, a DGT probe with a thin diffusion layer was used in order to get higher accumulation masses of trace metals. Such a type of DGT has been increasingly used for high-resolution study [37]. Previous studies have shown that the uses of thin diffusion layers down to 0.01 mm thickness do not differ in terms of DGT response from that of a conventional diffusion layer (0.90 mm) [38]. For this study, the binding gel impregnated with Chelex-100 resin (Bio-Rad, Hercules, CA, USA) was prepared based on Zhang and Davison [39]. When assembling the DGT probe, the Chelex gel was covered directly by a Durapore^®^ PVDF membrane (HVLP00010, Millipore, Shanghai, China) in a Perspex holder. The PVDF membrane has a pore size of 0.45 μm and a thickness of 0.10 mm, which was used as the thin diffusion layer. A sponge sheet was attached on the back of the Perspex holder, to identify the SWI location when retrieving the probe from the sediment after deployment [40]. These changes effectively facilitate field sampling with the DGT probe.

To prepare for the sampling event, the DGT probes were deoxygenated with nitrogen overnight and stored in a container filled with deoxygenated NaCl solution (0.1 M). DGT probes were inserted into the sediments using a customized releasing device [41] and deployed for 24 h. After retrieval, each probe was rinsed to remove visible sediment particles, and the SWI position was marked. The probes were placed in a sealed plastic bag at air temperature to prevent moisture loss and transported to the laboratory for analysis. The temperature of the overlying water was also recorded.

### 2.3. Sediment Sampling

The sediment cores at each site were collected using a gravity core sampler within a few minutes after insertion of the DGT device. Each core was sliced into 0.5 cm sections to a depth of 5 cm, and then into 1.0 cm sections from a depth of 5 to 10 cm. Each section was protected by high purity nitrogen gas. The sediment sections were lyophilized at −56 °C after cryopreservation, and then ground up to pass through a 100-mesh (0.15 mm) sieve. The samples were stored at 4 °C before analysis.

### 2.4. Sample Analysis

The Chelex binding gels were cut off at the marked SWI position; the gels were then removed from the DGT probes and vertically sliced into 3 mm sections. Each slice was transported into a centrifuge tube, and a 1.0 M HNO_3_ solution was added to elute the metals in the binding gel [35]. The Mn, Zn, Ni, Cu, Pb, Co and Cd concentrations were analyzed using an ICP-MS (NexION 300, Perkin-Elmer, Waltham, MA, USA), and the Fe concentration was analyzed using the phenanthroline colorimetric method with a Epoch Microplate Spectrophotometer (BioTek, Winooski, VT, USA).

Basic chemical properties of the sediment samples were analyzed using standard methods [42]. The total heavy metal concentrations in sediment samples were determined using microwave digestion with a mixture of HF–HNO_3,_ followed by ICP-MS or spectrophotometer analysis after approximate dilution. The pH value was measured in a deionized water suspension, at a solid/liquid ratio of 1:5 (5 g:25 mL, after 2 h of shaking and equilibration) using a pH electrode. The organic matter content in the sediments was measured in terms of total organic carbon (TOC) using a TOC analyzer (TOC-V CPN, Shimadzu, Kyoto, Japan).

Fractionation of metals was performed using a modified BCR method [43]. Briefly, a weight of 1.0 g dried sediment was added into 100 mL centrifuge tube and was sequentially extracted using 40 mL of 0.11 M acetic acid for 16 h, 40 mL of 0.5 M hydroxyammonium chloride for 16 h, 10 mL of 8.8 M hydrogen peroxide for 1 h (pH 2, repeat one time) and then 50 mL 1 M ammonium acetate (pH 2) for 16 h. The extracted metals were water and acid soluble (F1), reducible (F2), and oxidisable forms (F3), respectively. The residue after the four extractions was digested using aqua regia and the measured metals were residual form (F4).

### 2.5. Data Processing and Statistical Analysis

The DGT measurements were interpreted as time-averaged flux (*F*_DGT_) for each metal (pg·cm^−2^·s^−1^):

(1) $$F_{DGT}=\frac{M}{At}$$

where *M* is the mass (pg) of labile metals taken up by DGT. *A* is the exposure area of the gel (cm^2^), and *t* is the deployment time (s).

Relative mobility was used to assess the mobility of different metals, and is defined as the ratio of metals accumulated by DGT to the total metals in sediment:

(2) $$\text{Relative mobility}=\frac{\bar{M}\times A}{\bar{m}}\times 100\%$$

where $\bar{M}$ is the mean accumulation mass by DGT in vertical profiles (ng·cm^−2^). A is the exposure area of DGT probe in the sediment (cm^2^). The variable $\bar{m}$ is the mean total metal content in a volume of sediment (ng) with a thickness of 10 mm close to the surface of DGT probe. Modeling of DGT-induced diffusion showed that the metals accumulated by DGT mostly come from this thickness of sediment layer [44]. The $\bar{m}$ is calculated using total metal concentration, water content and sediment density.

## 3. Results and Discussion

### 3.1. Sediment pH and TOC

**Figure 2:**
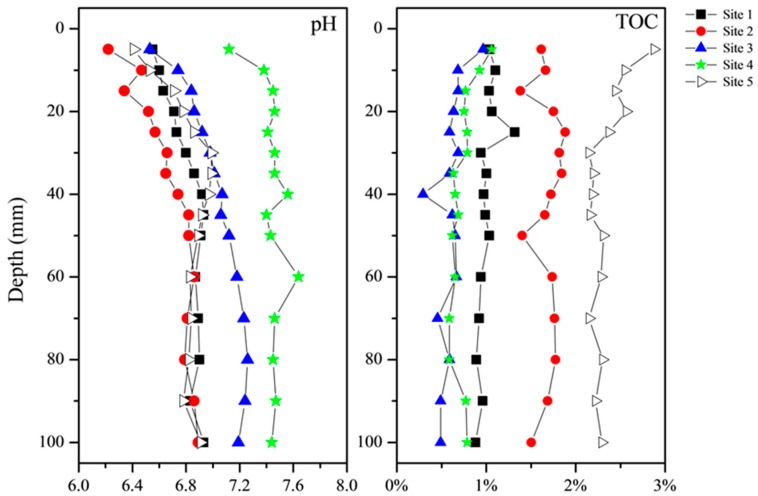
The distribution profiles of pH and TOC in sediments of five sites in Lake Taihu.

Sediment pH and TOC can influence metal lability, by changing the partition of labile forms [24,45,46]. Figure 2 presents the distribution profiles of pH and TOC in sediments of five sites. The pH value increased downward in surface sediments from the SWI to depths of 40, 50, 80, 20 and 30 mm at Sites 1 to 5, respectively, followed by a steady stage or a slight decrease until to the bottom of the profiles. The pH values at Site 4, ranging from 7.12 to 7.64, were evidently higher than those of other sites; the pH values at Site 3, ranging from 6.53 to 7.26, were followed after Site 4. Algal- and macrophyte-dominated regions showed similar pH variations across the profiles, with the pH values ranging from 6.22 to 6.99.

Macrophyte-dominated region at Site 5 had the greatest contents of TOC, followed by algal-dominated regions at Sites 1 and 2 and then transitional regions at Sites 3 and 4. The contents of TOC were on average 1.01%, 1.68%, 0.61%, 0.74% and 2.34% for Sites 1 to 5, respectively. Site 5 was also in an aquaculture region, where fresh aquatic vegetation and small aquatic animals are annually added in the water column to feed the crabs. Both the aquatic macrophyte decomposition and aquacultural activities contributed to the high content of TOC. The TOC had a downward decrease from 2.88% at the SWI to 2.37% at a depth of 25 mm at Site 5. Slightly downward decreases of TOC were also observed in surface sediments of Sites 3 and 4, while no evident trend appeared at other two sites.

### 3.2. Total Concentrations of Metals

**Figure 3:**
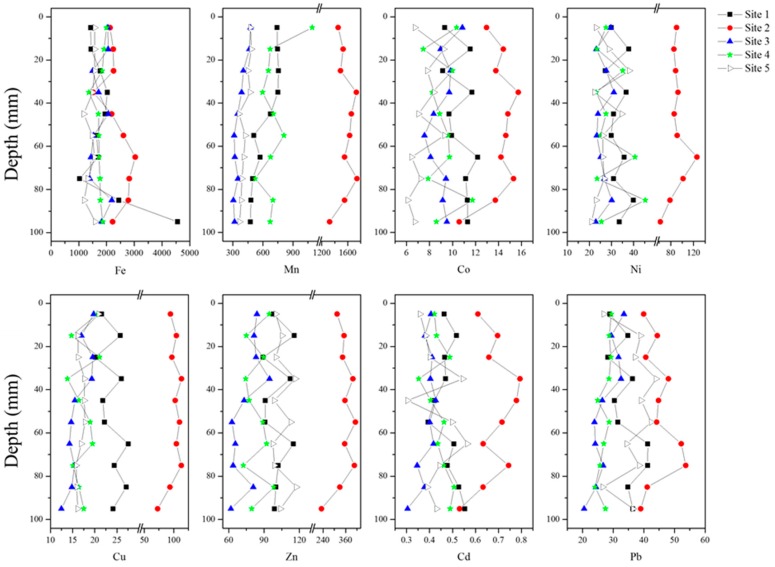
Distributions of total metal concentrations in sediments of five sites in Lake Taihu.

The vertical distributions of the eight metals in total concentrations are shown in Figure 3. Generally, the concentration of each metal kept stable with the depth of sediment. The relative standard error (RSD, *n* = 10) for each metal concentrations from the five sites are 18%, 16%, 12%, 18%, 14%, 17%, 12% and 24% for Fe, Mn, Zn, Ni, Cu, Pb, Co and Cd, respectively. All the metals showed a downward decreasing trend in Site 2 below the depth of 75 mm. Besides, Cd and Pb exhibited a downward decreasing trend at Site 3 below the depth of 35 mm.

Table 2 presents average total concentrations of the eight metals in the surface 10 mm sediments. The concentration ranges of the metals at the five sites were 19.42–35.60 (×10^3^, Fe), 360.9–1236.7 (Mn), 62.20–269.4 (Zn), 26.40–88.75 (Ni), 14.51–81.28 (Cu), 16.05–31.54 (Pb), 9.15–14.02 (Co) and 0.142–0.523 (Cd) mg·kg^−1^, respectively. The concentrations were highest for Fe and Mn, followed by Zn, Ni, Cu, Pb, and then Co, while Cd had the lowest values. Most of the metals showed higher concentrations in algal-dominated regions (Sites 1 and 2) than those in other regions (Sites 3–5). Site 2 had the greatest total concentrations for all the eight metals. This site is in Zhushan Bay which has been seriously polluted by sewage emissions and from agricultural non-point sources [14,47]. The higher concentrations of all the metals above the 75 mm depth may reflect a 35-year pollution history based on an average sedimentation rate of 2.1 mm·yr^−1^ in Taihu [10]. In contrast, Site 3 showed the least accumulations of Mn, Zn, Ni and Cd in sediments, reflecting that this site was much less influenced by anthropogenic activity. In addition, Sites 4 and 5 showed the least accumulations of Cu and Pb and Co in sediments, respectively.

### 3.3. Fractionation of Metals

**Figure 4:**
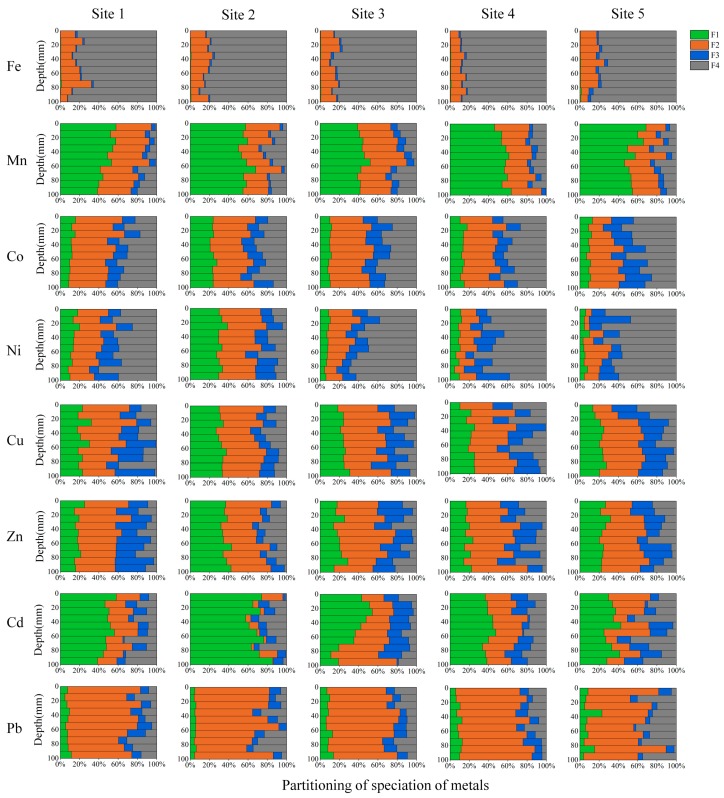
Speciation of metals in sediments based on a modified BCR scheme. The four forms are water and acid soluble (F1), reducible (F2), oxidisable (F3), and residual form (F4), respectively.

The metals were fractioned into four forms using the revised BCR procedure [43]. The speciation of each metal in the five sampling sites is present in Figure 4. The distribution of these forms could be divided into four types among the eight metals. The first type was Fe, which was dominated by residual form (F4). This form accounted for on average 81% to 86% of total Fe in sediments for the five sites, respectively. The acid-soluble form then contributed to on average 12% to 17% of total Fe. The dominance of the residual form reflected that Fe was much inert in sediment.

The second type was Mn and Cd, both of which were dominated by water and acid-soluble form (F1) followed by reducible (F2). In heavily-polluted Site 2, the extracted Cd was mostly composed by the water and acid-soluble form (accounting for 58%–85% of the total extracted Cd). In Site 3 containing the least total concentration of Cd, the water and acid-soluble Cd was dominated above the depth of about 30 mm, followed by a sharp decrease to the bottom. The contrast changes between the two sites reflected that the acid-soluble Cd was much labile and could reflect the contamination of Cd in sediment. This was consistent with the previous recognition by others [20,48].

The third type was Pb, which was dominated by reducible form (F2) in sediments. The reducible Pb accounts for on average 77%, 83%, 76%, 77% and 80% of the total extracted Pb in Sites 1 to 5, respectively. The proportion of residual form had a notable increase in Site 5 which contained the greatest TOC. Plach and Warren have revealed that the mobilization of Pb was controlled by natural organic matter (NOM) in NOM-rich freshwater sediments [49]. Since the reducible form of metals in BCR fractionation method was considered less labile than acid-soluble form [43], Pb in sediments of Lake Taihu should be much less labile in comparison to Cd.

Other metals (Co, Ni, Cu and Zn) showed an intermediate situation for the distribution of the four forms. The F1 and F4 were the major form for Ni and Co, while F1 and F2 were the major forms for Cu and Zn. In heavily-polluted Site 2, the contribution of F1 had an evident increase in relative to the residual form, reflecting an increase in lability of the four metals; In Site 5 containing the greatest content of TOC, the contribution of F3 and/or F4 for Co, Ni and Cu increased notably in relative to other sites, reflecting an increase in their inertness in sediments.

### 3.4. Vertical F_DGT_ Profiles of Metals

**Figure 5:**
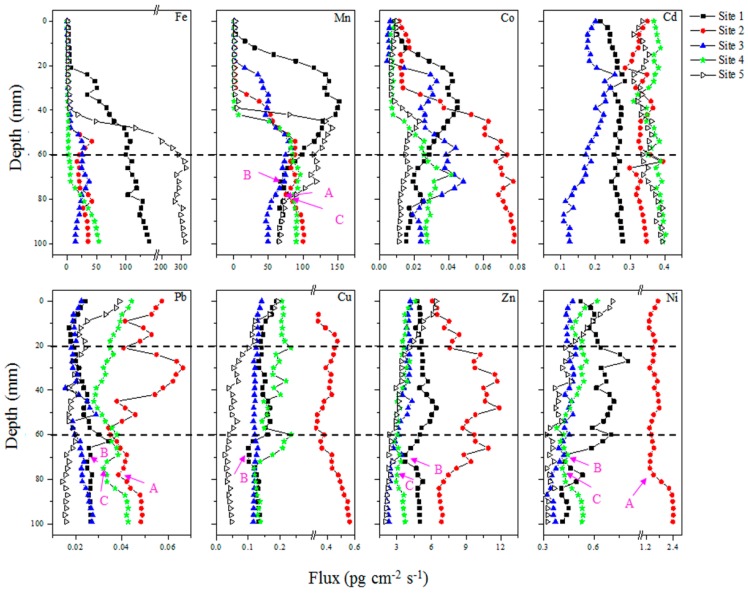
Distributions of DGT-labile flux (*F*_DGT_, pg·cm^−2^·s^−1^) of metals in sediments. The marked letters show corresponding minima between Mn and other metals.

Figure 5 shows the time-averaged fluxes (*F*_DGT_) of labile metals measured by DGT. The *F*_DGT_ of Fe remained steady with very low values in the surface sediment layers for the five sites investigated. Sites 1 and 5 had then a sharp increase consistently to the bottom. Other sites also had a sharp increase to a middle depth, followed by a steady stage (i.e., at Site 5) or a small fluctuation (i.e., at Sites 2 and 3). The depths showing the onset of the sharp increasing phase were 20, 42, 40, 72 and 40 mm for Sites 1 to 5, respectively.

The *F*_DGT_ of Mn at Sites 2 and 4 had similar profiles: they remained steady with very low values in the surface sediment layers, followed by sharp increases to middle depths and then remained steady to the bottom of the sediments. Sites 1, 3 and 5 also had a sharp increasing phase of *F*_DGT_ after a low-level stage in the surface sediment layer, followed by a downward decrease to the bottom of the sediment. The depths showing the onset of the sharp increasing phase of DGT-labile Mn were 10, 30, 18, 40 and 40 mm at Sites 1 to 5, respectively.

The distributions of DGT-labile Co were similar to those of Mn at the five sites. Sites 1, 3 and 5 showed an increase and then decrease after a low-level stable stage. Sites 2 and 4 showed the sharp increases of labile Co from middle depths followed by relatively steady stages to the bottom of the profiles.

The *F*_DGT_ value of Cd remained quite steady with the depth at Sites 1, 2, 4 and 5, except that a downward decrease appeared in the surface 20 mm layer at Site 2. Only Site 3 showed a considerable variation of *F*_DGT_ across the profile, reflected by an increase from the depth of 5 mm to 25 mm and then a consistent decrease to the bottom of the sediment.

The distribution of *F*_DGT_ for Pb showed considerable fluctuations with sediment depth. All the sites had a downward decrease in the surface 10–20 mm layer. Site 5 then remained steady to the bottom; Sites 1 and 3 had downward increasing trends to the bottom; Sites 2 and 4 had large variation with the depth, but both had visible minima with smaller *F*_DGT_ at the depth of 78 mm.

The distributions of *F*_DGT_ among Cu, Zn and Ni were similar, except for those at Site 2. The *F*_DGT_ had large decreases with depth in the surface 30 mm for the three elements, followed by slight decreases to the bottom of the profiles. Similar decreases in the surface sediments appeared for Cu at Site 1 and Ni at Site 4. Sites 1, 3 to 5 then remained relative steady with small fluctuations or had decreasing trends to the bottom with depth. Site 2 had different distributions of *F*_DGT_ among the three elements. The *F*_DGT_ of Cu and Ni showed a downward increase and decrease in the surface 20 mm and 10 mm, respectively, followed by a decrease and stable stage to a depth 57 mm and 78 mm. After that, they both had downward increases to the bottom of the profiles. The *F*_DGT_ of Zn had an increasing trend from the SWI to a depth of 36 mm, followed by a consistent decrease and then a stable stage to the bottom.

Visible minima with smaller values of *F*_DGT_ relative to their adjacent data appeared at the depth of around 60 mm at Site 1 for Pb, Cu, Zn and Ni (marked as B in Figure 5), at the depth of around 78 mm at Site 2 for Pb and Ni (marked as A in Figure 5), at the depth of around 54 mm at Site 4 for Pb, Zn and Ni (marked as C in Figure 5). Similar minima of *F*_DGT_ have appeared in the Mn profiles.

### 3.5. Overall Lability and Spatial Heterogeneity of Metals in Sediments

The measurement with DGT can reflect the lability of target analytes in sediments, since the measured species are two labile fractions of the analytes in sediments with high mobility, including dissolved fractions in pore water and easily-exchanged fractions released from sediment solids to resupply the pore water concentration due to DGT perturbance [31].

Table 3 presents the mean *F*_DGT_ and the relative standard deviations (RSD, *n* = 33) of the eight metals in vertical profiles across the five sites. A total of five metals (Zn, Ni, Cu, Pb and Co) had the greatest mean *F*_DGT_ at Site 2, which was consistent with their total concentrations in sediments (Table 2). Since the DGT-labile metals have been confirmed as bioavailable fractions with excellent exposure concentration response to toxic effects on aquatic organisms [50,51], the organisms at Site 2 located in Zhushan Bay may face elevated risks from exposure of the five metals with great mass accumulation and high lability in sediments [20].

The mean *F*_DGT_ at Site 1 to 4 was highest for Mn, followed by Fe, Zn, Ni, Cd, Cu, Pb, and then Co. This order was different from that of Fe, Mn, Zn, Ni, Cu, Pb, Co and Cd according to their total concentrations (Table 2), reflected by greater *F*_DGT_ values for Mn and Cd. It demonstrates that the sediments with lower total concentrations of Mn and Cd have stronger ability in resupply to pore water from the solid phase during the uptake of DGT. The Cd was generally considered as a labile element, and the sediment solids had been found to show a strong ability in resupply of porewater Cd in Esthwait Water, UK [32].

The variability (relative standard error, RSD) of the DGT-measured metal fluxes along the vertical direction was further used to assess the spatial heterogeneity in lability for each metal in sediments. The RSDs were in the ranges of 65.5%–154.1%, 50.3%–90.0%, 11.1%–38.5%, 11.4%–26.4%, 5.2%–64.6%, 14.5%–29.1%, 36.3%–59.3% and 5.5%–22.8% for Fe, Mn, Zn, Ni, Cu, Pb, Co and Cd, respectively. Fe, Mn and Co had the highest RSD values among the eight metals. As Fe and Mn were both redox-sensitive elements in sediments [52], the redox reactions, particularly those with Fe- and Mn-involved or controlled, should be vital factors influencing the variation of labile metals in sediments. The Cd had the lowest RSD values, reflecting that the lability of Cd remained relatively stable and should be less affected by redox reactions in comparison with other metals.

### 3.6. Relative Mobility of Metals in Sediments

The DGT accumulates labile fractions of metals in situ with high mobility in sediment [32,33,34]. The DGT-accumulated mass was used to demonstrate the relative mobility of metals in comparison with the total amounts of metals in a certain volume of sediment. Table 4 presents the relative mobility of eight metals at five sites in Lake Taihu. There was a large variation in the mobility among the eight metals. The relative mobility of Cd and Mn were the greatest, followed by Zn, Ni, Cu and Co, while Pb and Fe had the lowest mobility. The order was just consistent with the order arranged according to their distributions in chemical forms, especially the proportion of F1. Guevara-Riba et al. [48] studied the sediment mobility using the modified BCR approach, and defined mobility as the ratio of metals extracted in 0.11 M acetic acid (F1) to the sum of fractions; they also found that Cd had the greatest mobility in comparison with Zn, Pb, Cu, Ni and Cr. As the labile fractions of metals from chemical extraction are operationally defined with debate in reflection of their mobility [19,21,29], the DGT-derived indicator should be more accurate.

Seven of the metals (all except Cu) had the highest relative mobility in the samples from Site 5, which was different from the order assessed by F1. Site 5 is located in East Lake Taihu, and is famous for its aquaculture. The biological effects of crabs on the metals may have made the metals more labile, even though the total concentration was not the highest. As with the metals from Site 5, Cu at Site 4 and Pb at Sites 2 and 4 also deserve more attention because of their higher mobility, making these metals more labile and more easily accumulated in organisms.

### 3.7. Mechanisms for Fe, Mn and Co Remobilization

The results have shown a sharp increasing phase for DGT-labile Fe with depth in sediments (Figure 5). This increase phase has been observed with DGT measurements in both freshwater and seawater sediments [36,53]. It was attributed to the reductive dissolution of Fe (oxyhy)droxides under anoxic condition in deeper sediment layers and the resulted release and remobilization of Fe(II) [54,55]. The reducible Fe minerals in sediments are mainly ferrihydrite, lepidicrocite, goethite, hematite and akaganéite, while ferrihydrite and lepidicrocite may play a primary role in increasing the lability of Fe because they are more easily reducible in comparison with other Fe minerals [56,57].

Similar to the redox-sensitive Fe, the initial increasing phase of DGT-labile Mn was also attributed to reductive dissolution of Mn(IV) oxides. Both Mn(IV) oxides and Fe (oxyhy)droxides are major electron acceptors during the early diagenetic processes dominated by organic matter decomposition [52]. The depths showing the onset of the sharp increase of labile Mn were mostly shallower than those of Fe at four sites. It demonstrated that Mn(IV) oxides are more easily reduced than Fe (oxyhy)droxides, which is consistent with the terminal electron-accepting processes (TEAPs) in sediment where Mn(IV) reduction dominates followed by Fe(III) reduction [35,52]. On the other hand, oxidation of Mn(II) is slower than that of Fe(II), enable it to extend closer to the SWI if they contacts with oxygen. At Site 5, the depth showing the onset of the sharp increasing phase of DGT-labile Mn nearly equaled to that of labile Fe. A similar phenomenon has been observed in contaminated marine sediment. It was suggested that in strong reducing sediments, most released Mn(II) is adsorbed to freshly formed Fe oxides, rather than being oxidized [58]; The adsorbed Mn(II) can release again with Fe(III) reduction [45,58], causing simultaneous increases of DGT-labile Fe and Mn as observed.

**Figure 6:**
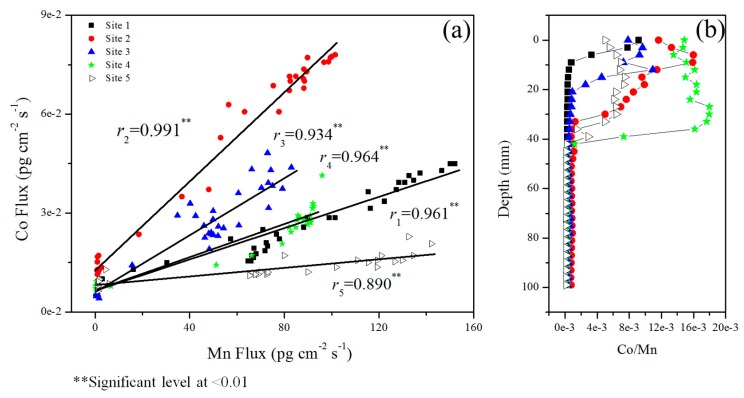
Correlation analysis between DGT-labile Co and Mn in sediments (**a**) and the changes of their ratio with sediment depth (**b**).

The distributions of labile Co were very similar to those of labile Mn at the five sites (Figure 5). This was further verified by positively significant correlations at very significant levels (*p* < 0.01) between them (Figure 6). Their precise correspondence in profiles has been reported in both freshwater [54,59] and marine systems [33,35,60,61,62]. Three mechanisms have been developed to explain their similarity in remobilization. (1) The reductive remobilization of Co and Mn required very similar redox conditions and they tended to occur simultaneously but independently; (2) Co may released from organic matter decomposition which supply electrons to cause the reduction of Mn(IV); (3) Co may be incorporated in Mn oxyhydroxides, and released with the reductive remobilization of Mn [35,61,63]. The last one should be the reason responsible for the similarity between the distributions of labile Co and Mn. It has been shown that Mn oxides had much greater preference than Fe oxides in trapping Co; Enrichment of Co within Mn oxides was 9 times that of Fe (oxyhydr)oxide from an investigation in marine sediment [62]. The presence of Co in individual Mn-oxide particles has also been verified by elemental analysis with electron microscopy [64]. And their association can be through sorption of Co(II) by Mn oxides [65]. Co(II) adsorbed to the oxide can be oxidized to Co(III) and subsequently incorporated into Mn oxide structure [66]. Co is then released from reductive dissolution of particulate Mn oxides, as verified in the water column of two freshwater lakes [63,65].

It should be noted that the dynamics of Mn(IV) reduction on controlling the remobilization of Co had a large variation among the 5 sites. The molar ratios of Co to Mn were on average 8.2 × 10^−4^, 3.7 × 10^−3^, 1.8 × 10^−3^, 6.1 × 10^−3^ and 2.3 × 10^−3^ for the Sites of 1 to 5, respectively. The values were similar to the Co/Mn ratio range of 1.1 × 10^−4^ to 2.6 × 10^−3^ reported by Lienamann et al. [64] from several studies of anoxic lakes. All the sites exhibited high values of Co/Mn ratio in the upper sediment layers followed by sharp decreases of the ratio (Figure 6). The depths at the end of these decreases generally corresponded to the onset of the sharp increases of labile Mn (Figure 5), reflecting that the reductive dissolution of Mn(IV) has caused the relatively greater increase of DGT-labile Mn in comparison to Co and the decreases of Co/Mn ratio. The molar Co/Mn ratios had a small variation after their sharp decreases, ranging from 1.4 × 10^−4^ at Site 5 to 7.6 × 10^−4^ at Site 2. The reason for this variation in deeper sediment layers is not clear. Stockdale et al. [62] performed an incubation experiment with intact and aged/homogenised marine sediments, and found that the ageing and homogenization of the sediments has caused the decrease of the Co/Mn ratio from 1.1 × 10^−3^ to 1.9 × 10^−4^. Co can be immobilized via formation of or incorporation into sulphide phases to a greater degree than Mn because that Co-sulphide phase is much lower soluble than Mn-sulphide phase. The variation of the Co/Mn may attribute to the immobilization of Co to a different degree via the formation of, or imcorporation into sulphide phases [62]. Further studies should be carried out to investigate the factors influencing the coupling between Mn and Co.

### 3.8. Mechanisms for Other Metal Remobilization

Previous studies have suggested three mechanisms responsible for the remobilization of trace metals at the SWI: (1) decomposition of organic material; (2) reductive dissolution of Fe/Mn (oxyhydro)xides; (3) desorption or adsorption at a pH gradient [32]. In this study, downward decreases in surface 10–20 mm sediment layer appeared for Pb at 5 sites, Ni at 2 sites and Cu and Pb at 1 site. This phenomenon was seldom reported in previous studies. These decreases could not be attributed to the decomposition of organic matter, which has been found to cause downward increases of metals and pronounced surface maximum of DGT-labile metals nearby the SWI [1,24,35,45]. In addition, the decomposition of organic matter was not notable at Sites 1 to 4, reflected by the relatively stable distributions of TOC (Figure 2). The effect of reductive dissolution of Fe/Mn (oxyhydro)xides was also excluded. This process should induce the remobilization of trace metals and the increase of DGT-measured flux/concentration in surface sediments as observed previously in the Rupel River, Belgium [54] and in a sulphidic freshwater [67]. The decreases of labile metals may attribute to the downward increases of pH values in the surface layer at the five sites. The increase of pH could promote the precipitation of metals and decrease the dissolution of metals, resulting in the decreases of DGT fluxes as observed. The vertical decreases in totally four metal fluxes at Site 5 were accompanied by the largest decrease of the pH value, while other sites only showed the decreases of 1–2 metals. This difference could partly support the above hypothesis.

There were several corresponding minima of *F*_DGT_ between Mn and metals at and below the depths of 60 mm (Figure 5). The correspondences were more evident for Pb and Ni, for which three sites appeared this phenomenon. It was followed by Zn and Cu, for which two and one sites appeared this phenomenon. It reflects that the remobilizations of Pb, Ni, Zn and Cu in the deep sediments may to a certain degree relate to Mn cycling. As sulphate is the primary electron acceptor in deeper sediment layer when the reactive Fe and Mn oxides are consumed, the elevated sulphide production may be a major mechanism in removal of Mn and the four metals via sulphide precipitation. Yin et al. [68] reported that the concentration of acid volatile sulfide (AVS) in the sediments of Lake Taihu were 5.09 μmol·g^−^^1^ and 2.94 μmol·g^−^^1^ in the sediments of two sites in Meilang Bay, Lake Taihu. The ASV showed sharp downward increases below the depth of 6 cm and had maxima at the depths of around 8 cm. The depths with the maxima generally corresponded to the depths appearing the localized minima of *F*_DGT_ for Mn and the four trace metals.

The vertical distributions of metal fluxes at the middle depths had no detailed spatial structure. The release of metals may from various sources, and two or more mechanisms should work separately or together to determine the remobilization of metals. Simultaneous measurements of metals and potentially governing factors at a fine scale using DGT and other high-resolution methods (e.g., planar optode) may greatly facilitate the discovery of those mechanisms.

**Figure 7:**
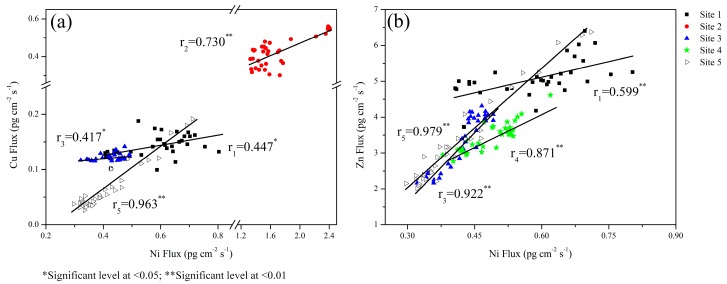
Correlation analyses of labile Ni with labile Cu (**a**) and labile Zn (**b**) in sediments.

Positive correlations were obtained for labile Ni with labile Cu and with labile Zn at totally 4 sites (Figure 7). Similar correspondence of Ni with Cu and Zn had been observed from a local remobilization associated with macrofauna in a deeper sediment layer of the North-East Atlantic [33]. It demonstrates that the three elements had a close link between the geochemical behaviors, including similar source materials and recycling processes.

## 4. Conclusions

The mean *F*_DGT_ at four sites was the highest for Mn, followed by Fe, Zn, Ni, Cd, Cu, Pb, and then Co. The variability (RSD) of the *F*_DGT_ along the vertical direction were much greater for Fe, Mn and Co than for the other metals, reflecting that the redox reactions were vital factors influencing the variation of labile metals in sediments. The relative mobility, which was calculated based on the use of *F*_DGT_, followed the order of Cd, Mn, Zn, Ni, Cu and Co, Pb and Fe. Cadmium in sediments deserved more attention because of its high mobility. Both DGT-labile Fe and Mn had sharp increasing phases with depth in sediments. The depths showing the sharp increase of labile Mn were mostly shallower than those of Fe at four sites, demonstrating that Mn(IV) oxides are more easily reduced than Fe (oxyhy)droxides. Both the depths were almost the same at Site 5, likely reflecting that in strong reducing sediments most released Mn(II) is adsorbed to freshly formed Fe oxides, rather than being oxidized. The distributions of labile Co were very similar to those of labile Mn at the five sites, with the Co/Mn ratios of 8.2 × 10^−4^, 3.7 × 10^−3^, 1.8 × 10^−3^, 6.1 × 10^−3^ and 2.3 × 10^−3^, respectively. It demonstrated that Co should be incorporated in Mn oxyhydroxides, and released with the reductive remobilization of Mn, while the variation of the Co/Mn was attributed to the immobilization of Co to a different degree via the formation of, or incorporation into sulphide phases. Downward decreases in surface 10–20 mm sediment layer appeared for labile Pb, Ni and Cu at several sites, which were attributed to the downward increases of pH values in the same depth. The remobilizations of labile metals in deep sediment layer, together with several corresponding minima with Mn, may attribute to coprecipitation by elevated sulphide. In addition, Ni, Cu and Zn had a close link in their geochemical behaviors, reflected by the positive correlations of labile Ni with labile Cu and Zn.

## Figures and Tables

**Table 1:** Location of sampling sites in Lake Taihu.

Site	Longitude	Latitude	Description
1	120°8′45.24″	31°24′24.48″	In Meliang Bay; algae-dominated
2	120°2′42.12″	31°27′0.04″	In Zhushan Bay; algae-dominated
3	120°10′48.72″	31°14′17.99″	In the central part of the lake
4	120°4′36.12″	31°5′24.36″	In the west part of the lake
5	120°30′47.88″	31°5′21.88″	In East Taihu; macrophyte-dominated

**Table 2:** Mean total metal concentrations (mg·kg^−1^) in sediments of five sites in Lake Taihu ^a^.

Metal	Sampling Sites				
	1	2	3	4	5
Fe × 10^3^	2.00	**2.38**	1.78	1.77	1.46
	1.03–4.56	1.52–3.04	1.40–2.19	1.37–2.00	1.18–1.60
Mn × 10^3^	0.62	**1.57**	0.37	0.71	0.42
	0.48–0.75	1.32–1.74	0.31–0.48	0.52–1.09	0.36–0.48
Zn	101	**350**	75.1	84.2	105
	89.2–116	232–412	61.8–94.6	72.5–98.3	97.4–118
Ni	33.3	**90.4**	26.4	29.7	27.0
	27.2–39.9	62.0–126	22.9–31.1	23.1–45.3	21.0–38.1
Cu	24.1	**101**	16.3	17.4	17.1
	20.1–27.6	72.7–113	12.5–19.8	13.8–21.1	15.8–20.9
Pb	34.4	**44.8**	27.3	27.4	36.5
	28.2–41.3	39.0–53.7	20.5–33.5	24.1–29.4	26.6–44.0
Co	10.73	**14.02**	9.15	9.25	7.50
	9.14–12.2	10.6–15.7	7.57–10.9	7.46–11.7	6.12–9.31
Cd	0.48	**0.68**	0.39	0.45	0.43
	0.39–0.55	0.53–0.79	0.30–0.43	0.35–0.51	0.30–0.56

^a^ Highest values among different sites are marked in bold.

**Table 3:** Mean *F*_DGT_ (pg·cm^−2^·s^−1^) and RSD (%) of eight metals in vertical profiles at five sites in Lake Taihu ^a^.

Site	1		2		3		4		5	
	Mean	RSD	Mean	RSD	Mean	RSD	Mean	RSD	Mean	RSD
Fe × 10	7.51	65.5	1.55	95.0	1.28	93.0	1.16	154.1	**14.61**	96.5
Mn × 10	**8.60**	50.3	5.31	76.2	4.48	59.2	4.80	90.0	5.98	89.3
Zn	5.05	11.1	**8.63**	20.3	3.34	24.0	3.46	11.8	3.32	38.5
Ni × 10^−1^	5.82	19.1	**16.73**	20.8	4.23	12.5	4.86	11.4	4.16	26.4
Cu × 10^−1^	1.41	15.0	**4.21**	19.2	1.22	5.2	1.73	21.2	0.68	64.6
Pb × 10^−2^	2.40	15.2	**4.77**	18.4	2.19	14.9	3.60	14.5	2.02	29.1
Co × 10^−2^	2.63	42.9	**4.85**	56.0	2.54	51.8	1.84	59.3	1.19	36.3
Cd × 10^−1^	2.62	5.6	3.33	6.2	1.78	22.8	**3.72**	5.5	3.49	6.8

^a^ Highest mean among different sites is marked in bold.

**Table 4:** Relative mobility (%) of eight metals at five sites in Lake Taihu ^a^.

Elements	Sampling Sites				
	1	2	3	4	5
Fe	0.03	<0.01	<0.01	<0.01	**0.09**
Mn	1.21	0.42	1.05	0.64	**1.66**
Zn	0.62	0.56	0.61	0.66	**1.20**
Ni	0.15	0.24	0.24	0.36	**0.49**
Cu	0.10	0.06	0.11	**0.15**	0.08
Pb	0.01	**0.02**	0.01	**0.02**	**0.02**
Co	0.05	0.07	0.05	0.04	**0.08**
Cd	5.00	3.31	4.09	5.43	**9.77**

^a^ Highest mobility among different sites is marked in bold.
